# Subsampling effects in neuronal avalanche distributions recorded *in vivo*

**DOI:** 10.1186/1471-2202-10-40

**Published:** 2009-04-29

**Authors:** Viola Priesemann, Matthias HJ Munk, Michael Wibral

**Affiliations:** 1 Department of Neurophysiology, Max Planck Institute for Brain Research, Deutschordenstrasse 46, D-60528 Frankfurt am Main, Germany; 2 Group for Neural Theory, DEC, Ecole Normale Supérieure, Collège de France, 3, rue d'Ulm, 75005 Paris, France; 3 Deptartment of Physiology of Cognitive Processes, Max Planck Institute for Biological Cybernetics, Spemannstrasse 38, D-72076 Tübingen, Germany; 4 MEG Unit, Brain Imaging Centre, J.W. Goethe University, Heinrich Hoffmann Strasse 10, D-60528 Frankfurt am Main, Germany

## Abstract

**Background:**

Many systems in nature are characterized by complex behaviour where large cascades of events, or avalanches, unpredictably alternate with periods of little activity. Snow avalanches are an example. Often the size distribution f(s) of a system's avalanches follows a power law, and the branching parameter sigma, the average number of events triggered by a single preceding event, is unity. A power law for f(s), and sigma = 1, are hallmark features of self-organized critical (SOC) systems, and both have been found for neuronal activity *in vitro*. Therefore, and since SOC systems and neuronal activity both show large variability, long-term stability and memory capabilities, SOC has been proposed to govern neuronal dynamics *in vivo*. Testing this hypothesis is difficult because neuronal activity is spatially or temporally subsampled, while theories of SOC systems assume full sampling. To close this gap, we investigated how subsampling affects f(s) and sigma by imposing subsampling on three different SOC models. We then compared f(s) and sigma of the subsampled models with those of multielectrode local field potential (LFP) activity recorded in three macaque monkeys performing a short term memory task.

**Results:**

Neither the LFP nor the subsampled SOC models showed a power law for f(s). Both, f(s) and sigma, depended sensitively on the subsampling geometry and the dynamics of the model. Only one of the SOC models, the Abelian Sandpile Model, exhibited f(s) and sigma similar to those calculated from LFP activity.

**Conclusion:**

Since subsampling can prevent the observation of the characteristic power law and sigma in SOC systems, misclassifications of critical systems as sub- or supercritical are possible. Nevertheless, the system specific scaling of f(s) and sigma under subsampling conditions may prove useful to select physiologically motivated models of brain function. Models that better reproduce f(s) and sigma calculated from the physiological recordings may be selected over alternatives.

## Background

The brain is a complex dynamical system that consists of up to 10^11 ^neurons. These neurons interact in a mainly local and non-linear way and generate activity, which exhibits two seemingly contradictory properties: First, activity levels fluctuate over many orders of magnitude in time and space [1-8] even when external conditions are held as constant as possible [9-11]. And second, operation is stable enough to ensure brain function throughout its lifetime.

The combination of these two properties – fluctuations over many orders of magnitude and long-term stability – is also observed in a wide range of dynamical systems in nature, such as the interplay of geological plate tectonics and earthquakes, snowfall and snow avalanches, or tree growth and forest fires. In these systems, activity typically spreads via local connections and forms cascades of events, called avalanches. In particular systems, among them earthquakes, forests fires and avalanches in piles of rice, the distribution *f(s) *of an appropriate spatial or temporal measure, e.g. the avalanche sizes *s*, follows a power law [10,12-14]:

(1.1)

where τ is the system specific exponent. Such a power law distribution of avalanche sizes was also found in a simple model based on local and non-linear interactions introduced by Bak and colleagues [15]. They termed the mode of operation of this system 'self organized critical' (SOC), to reflect that their system showed fluctuations like physical systems at the critical point [e.g. [16], as discussed in [17]] and would converge to this state without fine tuning of parameters, i.e. self-organized. Bak and colleagues proposed SOC as a generic mechanism that generates the power law behaviour which is often observed in nature.

In many SOC systems with avalanche like activity, one can also define a branching parameter σ as the average number of subsequent events that a single preceding event in an avalanche triggers [2,18]. If the branching parameter can be defined in a SOC system, it is assumed to equal unity.

Operation in the SOC state has also been proposed as an explanation for the presence of large fluctuations, the susceptibility to small perturbations and the long term stability in brain activity [2,4-6,19-28]. In addition, theoretical investigations demonstrated that the critical state was optimal for information transmission and processing [29,30]. *In vitro *experiments provided first evidence for this "SOC hypothesis" by showing that avalanche sizes of suitably defined neuronal events approximately followed a power law in slice cultures of rat cortex [2]. Whether the SOC hypothesis is supported by experimental results *in vivo *is currently under debate [4,31]. This may partly be due to the problem that the theory of SOC does not provide a set of conditions that is sufficient for the classification of a system as SOC [17]. Past studies focused mainly on one hall mark feature of SOC systems – the power law distribution for the avalanche size or duration, or for the decay of temporal correlations [2,6,7,24,31,32]. Note however, that non-SOC systems may also show power law distributions [33,34].

Here, we hypothesize that it is even difficult to assess whether avalanche sizes in brain activity are distributed according to a power law, because brain activity is typically spatially subsampled. For example, in electrophysiological experiments, only the activity of a small fraction of the brain can be sampled simultaneously. It has not been investigated yet how subsampling affects the results of criticality analyses, i.e. avalanche distributions and branching parameters, and whether subsampling effects depend on the system under investigation. This theoretical gap challenges current attempts to classify brain activity as being in the SOC state.

It may be important at this point to highlight the differences between subsampling as it was investigated here and scaling analysis of SOC systems [35-38]. Scaling analysis deals with deviations from power law distributions, which are caused by the finite size of the system. However, for scaling analysis the system is *fully *sampled. In contrast, subsampling deals with the statistics obtained when sampling only a *small part *of the system. It has previously been demonstrated in the context of scale-free networks, that the effects of subsampling may be very different from those of scaling the system, and that they may fundamentally change the observed statistics [39].

In the present study, we investigated the effects of subsampling in three different SOC models, to derive estimates for the avalanche distributions that are to be expected in subsampled SOC systems. We also investigated the influence of subsampling on the branching parameter σ.

We compared these results to those calculated from local field potential (LFP) activity recorded in the behaving monkey. We found that, upon subsampling the SOC models, f(s) did not show a power law and σ was smaller than unity. Both, f(s) and σ, depended sensitively on the subsampling geometry and the dynamics of the model. Only one of the SOC models, the Abelian Sandpile Model (ASM) [15] which is characterized by local and recurrent activity, exhibited f(s) and σ similar to those calculated from LFP activity.

## Methods

### Models

We wanted to investigate subsampling effects in SOC systems. This necessitates a choice of mathematically well understood SOC models, where subsampling effects can be distinguished from finite size effects and where the corresponding finite size scaling has been quantified [15,36,38,40,41].

#### Abelian Sandpile Model (ASM)

We simulated the ASM [15] on a two dimensional square grid of size L^2 ^with L = 50 grid units (g.u.). In this model, each site of the grid carries an integer number of sand grains z(x, y) ≥ 0. If a site carries more than a critical number z(x, y) ≥ z_c _= 4 of grains it is called unstable and topples. Its height is reduced by 4 and each of its four next neighbours receives one grain. In the subsequent time step, all sites that became unstable in the previous one, topple simultaneously. The toppling activity propagates over the grid and forms so called avalanches. The size of an avalanche is defined by its total number of topplings. If all sites of the system are stable again, a grain is added to the system at a random site. This then may or may not initiate a new avalanche, depending on the state of the site before the addition of the grain. Grains that topple over the boundaries are lost from the system (dissipation; the reason for finite size effects).

#### Random Neighbour Model (RNM)

The RNM [41] is a variation of the ASM, where the grains from an unstable site are distributed not to the next neighbours, but to randomly chosen sites. We simulated the RNM on a grid of size L^2 ^L = 50 g.u.. Here, no boundaries were present. Thus, to implement dissipation, grains were removed from the system at random sites with probability r = 0.010923. The probability for dissipation r was calculated from the ASM:



The factor 4 in the denominator is due to 4 toppled grains per toppling. This probability is smaller than the ratio



because more topplings occur in the center of the ASM than at its borders.

#### Forest Fire Model (FFM)

The two-dimensional FFM [40] was calculated on a square grid of size L^2 ^with L = 50 × 50 g.u.. Each site is either occupied by a tree z(x, y) = 1, or empty z(x, y) = 0. A fire is initiated by a lightning that strikes a random site. If that site is occupied by a tree, it burns down. In the subsequent time steps, each tree that was next neighbour to a burning tree burns down, until the fire has burnt down the complete cluster of trees. The size of the fire (i.e. the avalanche) is the total number of trees that burnt down. After a fire, trees are grown on random sites with probability p = 2.4%.

In the ASM, RNM and FFM, the time between subsequent avalanches is defined to be infinite (infinite separation of time scales) [15,40,41].

#### Subsampling

**Figure 1:**
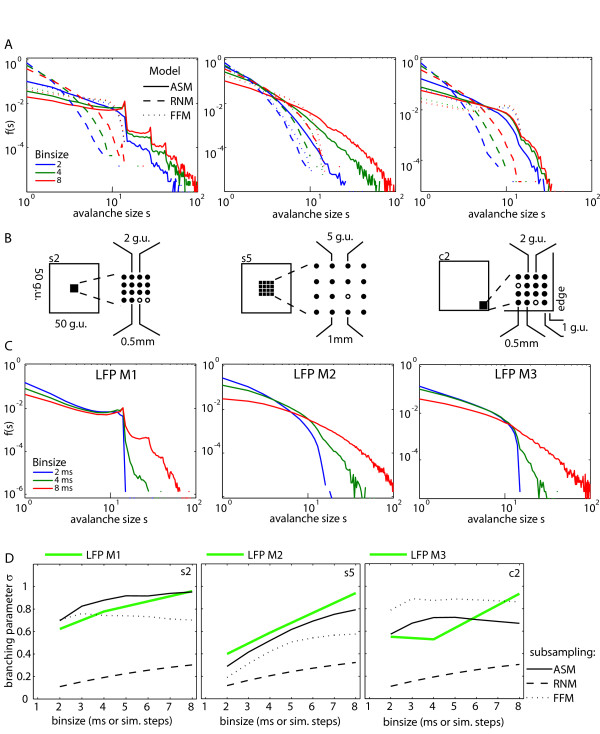
**Comparison of subsampling effects in avalanche distributions and branching parameters from model systems and *in vivo *LFP data**. (A) Avalanche distributions calculated from events sampled on a small fraction of the model sites (as indicated in B). None of the models shows a power law for f(s). Note that the characteristic peaks in f(s) are only expressed in the ASM and only when the distance between the sampling sites is small (left column). Avalanche distributions of the ASM (full lines), the RNM (dashed lines) and the FFM (dotted lines) are plotted. The colours indicate the different bin sizes (blue 2 steps; green 4 steps; red 8 steps). (B) Recording electrode configurations and corresponding sampling sites used in the simulations. The circles indicate the position of the electrodes, full circles indicate the electrodes that provided data for the evaluation of the LFPs. The inter electrode distance is given at the bottom of each figure. The full circles at the same time indicate the configuration of the subset of sampling sites sampled in the models. The left part of each figure indicates the position of the subsets of sampling sites with respect to the grid the model was simulated on. The left figure indicates the subset of sampling sites s2: 4×4 sites with distance 2 grid units (g.u.) between the sites, located in the center of the grid. The middle figure 4 enotes the subset of sampling sites s5, and the right the subset of sampling sites c2. (C) Avalanche size distributions f(s) for the binary events calculated from the LFPs. The colours indicate the bin size (blue 2 ms; green 4 ms; red 8 ms): left figure – Monkey1 (LFP M1), middle – Monkey2, LFP2 M2, right – Monkey3, LFP M3. The corresponding electrode configurations are plotted in part (B). (D) Branching parameter σ over bin size (in ms for LFP data and steps for simulated data) for the subsampled models and the LFP data. (Left) σ for sampling on subset s2 (related to the LFP recording geometry in M1). Green, solid line: σ for LFP of M1; dashed lines: σ for the subsampled ASM, FFM, and RNM; (Middle) σ for sampling on subset s5 (LFP of M2), same colour codes; (Right) σ for sampling on subset c2 (LFP of M3), same colour codes.

We sampled the activity (topplings or burnt trees) on the whole grid as well as on selected subsets of sampling sites. The choice of the subsets coarsely resembled the geometry of the electrode positions in the brain used in our electrophysiological recordings (see section on experimental data and figure 1B). We sampled 4×4 sites with varying distance *d *between the sites. The subsets of sampling sites were located either in the centre or next to a corner of a grid. If any of the 16 electrodes in an experiment did not contribute to the activity, we created a subset of sampling sites that was modified accordingly. Here, we present three subsets of sampling sites for which sampled activity provided the best match with the experimental results:

s2: 4×4 centred sites with *d *= 2 g.u. and 2 sites removed (figure 1B, left)

s5: 4×4 centred sites with *d *= 5 g.u. and 1 site removed (figure 1B, middle)

c2: 4×4 sites with *d *= 2 g.u. located with distance 1 g.u. to a corner of the grid and 2 sites removed (figure 1B, right)

### Experimental Methods

#### Experimental Setup

The animal experiments were performed according to the German Law for the Protection of Experimental Animals. The procedures also conformed to the regulations issued by the NIH and the Society for Neuroscience.

In order to assess whether subsampling effects could be identified in neuronal data – that necessarily come from extremely sparsely sampled systems – we recorded local field potentials (LFPs). LFPs were recorded simultaneously from up to 16 microelectrodes in three macaque monkeys (M1: female, 6 kg; M2: male, 12 kg: M3: female, 8 kg). Electrodes were located in the ventral prefrontal cortex (PFC) in two monkeys (M1, M2) and in the dorsolateral PFC, directly dorsal of the principal sulcus in one monkey (M3). The electrodes had impedances between 0.2 and 1.2 MΩ, at 1 kHz, and were arranged in a square grid with inter electrode distances of either 0.5 or 1.0 mm. For these spacings, LFP signals at each electrode are dominated by sources close to that electrode. A quantification of signal crosstalk due to volume conduction would require knowledge of the tissue conductivity tensor. However, in the far field limit that applies to our recordings, the signal tapers off proportional to (1/r)^n ^with n ≥ 2 leading to a dominance of local sources [42,43]. Signals were amplified and filtered (5–150 Hz) before being digitized at 1 kHz. The monkeys performed a visual short term memory task with on average 80% correct behavioural responses which required them to memorize a sample object and to compare a test stimulus presented after a delay of three seconds to memory content The monkeys indicated via differential button press whether test and sample stimuli matched or not. Each trial consisted of a 1000 ms long baseline, 500 ms sample stimulus presentation, a delay of 3000 ms and a response interval lasting throughout a 2000 ms test stimulus presentation. We used data from three experimental sessions containing a total of 3418 trials. Details of the experimental procedure can be found in [44].

### Analysis Methods

#### Event Definition

**Figure 2:**
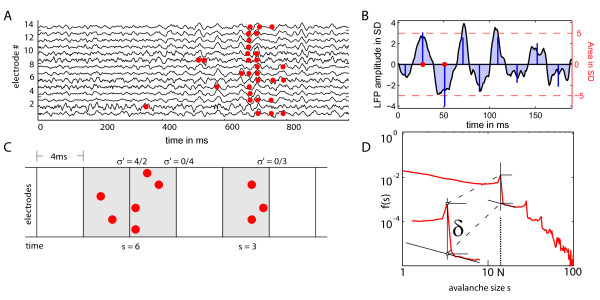
**Definition of binary events from raw LFP data and detection of avalanches**. (A) Sample LFP traces recorded simultaneously on 14 electrodes in monkey 1. LFPs were z-transformed with respect to pre-trial baseline. The red dots indicate binary events calculated from the LFP traces (see figure part B). (B) Algorithm for calculating binary events from the LFPs. Deflection lobes under the LFP trace are coloured in grey. Blue bars indicate the value for the area under a deflection lobe between two zero crossings. A binary event (red dot) is generated, if the absolute value of the area exceeds a threshold of 5SD of the absolute areas of deflection lobes in the baseline. (C) Avalanche definition. Binary events are concatenated in temporal bins (here: 4 ms). The avalanche size *s *is the total number of events in subsequent nonempty time bins. The single-step branching ratio σ' for the transition from one time bin to the next is calculated as described in the methods. The branching parameter σ is defined as the average of all single-step branching parameters. (D) Definition of the drop parameter δ for avalanche distributions with peaks at the number of sampled sites/electrodes and its multiples (N, 2N, ..). The drop delta is the difference of the value of f(s) at N and the value at this point obtained by linear extrapolation from the right (see methods).

While binary events are at the heart of our simulations and inherent in the 'all- or- none' generation of action potentials, analogue data like LFP recordings have to be transformed into binary events before SOC analysis can proceed. Raw analogue values of each LFP channel were z-transformed with respect to the variance of their pre-stimulus baseline, determined across all trials of each experiment (figure 2A). Then binary events were obtained from the analogue and continuous LFP data: In order to define a binary event in the LFP data, we calculated the *absolute *value of the area under a deflection lobe between two zero crossings. This measure is a function of the net sum of displaced ionic charges caused by the underlying synaptic events. If this measure exceeded a threshold of 5 SD of the baseline values, a binary event was added (figure 2B). We chose this threshold such that the rate of events was comparable to that of spiking activity in similar recordings from prefrontal cortex [44].

#### Avalanche distributions

The binary events obtained from models and experiments (a toppling or burnt tree in the model, a binary event calculated from the LFP traces) formed spatio-temporal clusters, called avalanches. For further evaluation, we partitioned the time axis into discrete bins. The avalanche size s was then defined as the total number of events in subsequent nonempty time bins (figure 2C). The size of the time bins was varied systematically [2]. For each bin size T the frequency distribution f(s) of avalanches of size s was calculated.

Variations in the bin size T applied to activity sampled on subsets of sampling sites were expected to change the avalanche size distribution f(s), because avalanches may leave a subset and at a later point in time reenter that same subset. This way, a single avalanche is counted as being two avalanches in this subset of sampling sites. Only if T exceeded the time between leaving and reentering, the avalanche would be concatenated again. Thus larger bin sizes were expected to result in more large and less small avalanches in *subsampled *systems.

The avalanche distributions f(s) obtained from our analyses depended on several independent variables such as data type (LFP events, model events), experiment/monkey or model system and subsampling geometry. Whenever necessary, these parameters are indicated in brackets of f(s) as demonstrated in the following examples: The avalanche distribution calculated from the LFPs recorded in monkey 1 (M1) is denoted by f(s; LFP, M1), and those calculated from the ASM on the subset of sampling sites s2 are denoted by f(s; ASM, s2).

#### Normalization

All avalanche distributions f(s) were normalized to the total number of elementary events (topplings, burnt trees, LFP events) that were recorded or sampled in the experiments or simulations, respectively:

(1.2)

F(s) denotes the avalanche distribution before normalization. A normalization to unity on the interval *s *∈ [1;∞] as it is required for a *probability *distribution is impossible for a power law distribution with exponent τ ≤ 1 due to the divergence of the integral.

#### Branching Parameter

The branching parameter (σ) is defined in the theory of branching processes [45]. σ represents the average number of events triggered by a single event in the previous time step within an avalanche (figure 2C). It is defined as the number of ancestors per predecessor averaged over all time steps t in all avalanches n:

(1.3)

N is the total number of avalanches, t_on_(*n*) is the time step of the first event in avalanche *n*, t_off_(*n*) is the time step of the last event in avalanche *n*, and a(t) is the total number of events at time step t.

For a branching process, the expected size of generation k equals σ^k^. Consequently, in the *sub*critical state, where σ is *smaller *than unity, any avalanche will die out within finite time. In the *super*critical state, where σ is *larger *than unity, some of the avalanches may become infinite. The probability for large avalanches is increased compared to the subcritical state. In the critical state, σ equals unity, and the avalanche size distribution follows a power law, thus the system is scale free: The avalanches do not have any characteristic size.

Here, we calculated σ for the binary data obtained from the LFPs, and for all subsets of sampling sites of all models.

#### Assessment of similarity between experimental and model avalanche distributions

We wanted to determine whether certain subsampled systems would approximate the experimental data better than others. Therefore, we quantified the similarity between the avalanche distributions obtained from models and those obtained from *in vivo *data on the interval 1 ≤ s ≤ 2·N where N is the number of recording sites. The measure for the dissimilarity of the distributions, the dissimilarity factor DF, was defined as standard deviation of the difference between the logarithm of the model and of the experimental distributions for bin size 4 and 8 time steps or ms, respectively. This measure is independent of an arbitrary choice of normalization:

(1.4)

Where  is the standard deviation taken over all avalanche sizes s. This dissimilarity factor DF is related to the Kullback-Leibler divergence (KLD) of two probability distributions *f*_1 _and *f*_2_[46]:

(1.5)

Since in our case most of the information is in the tail of the distributions where f(s) tends to be small, we removed the factor *f*_1_(*s*) that weighs the differences between the two distributions by the first one and consequently gives too much significance to the initial interval of the distribution in our case. Moreover, we knew that scaling was arbitrary, hence we were not interested in the absolute distance of the distributions (as measured by the sum or integral of log(*f*_1_(*s*)/*f*_2_(*s*)) over all values of s). If the two distributions *f*_1_(*s*) and *f*_2_(*s*) are identical apart from a scaling factor – which is the best we can hope for when comparing avalanche distributions – the fraction inside the integral of the KLD should be a constant. If distributions vary not only by scaling factor, this fraction will be variable. To estimate this variability we chose to use the standard deviation in equation (1.4). Hence, a small value for DF indicates that distributions are similar up to a scale factor.

We calculated DF for all possible combinations of f(s) from the experiments and the critical models sampled on different subsets of sampling sites. The minimum value of the computed DFs indicated the best match between an experimental and a model distribution.

To enable the computation of the logarithm for our simulated data where certain large avalanche sizes do not appear in the simulations – due to limited simulation runtime -, zero values in the distribution f(s) were substituted by 0.1/c to obtain real numbers only. This replaces the smallest possible avalanche frequency, 0, by a number that is still smaller than the next smallest possible avalanche frequency, 1/c and does not change the ordering of results. Here c is the normalization constant defined in equation (1.2).

To demonstrate that a model selection is feasible in principle by comparing avalanche distributions obtained from subsampled models and the experimental data we compared the dissimilarity factor (DF) between the data and various combinations of model systems (ASM, RNN and FFM) and subsampling geometries (s2, s5, c2).

#### Statistical Tests

Jensen stated in his book 'Self-Organized Criticality' [17] that local interactions of non-linear threshold units are essential to build a SOC system. We wanted to test the dependency of the observed avalanche distributions on these local interactions. More specifically, we wanted to demonstrate that these interactions were necessary for the appearance of peaks (cf. results section) in the avalanche distribution observed in the LFP data and in the ASM when s2 was sampled. In this context, peaks were quantified by a drop δ at s = N (figure 2D). We calculated the drop δ at the total number N of electrodes (or sites of a subset of sampling sites) as the difference between the peak value f(N) and the extrapolation of an approximation function g(s) evaluated at s = N.

(1.6)

The function g(s) was assumed to follow a power law with exponent -1, g(s) ~ s^-1^. It was fitted to the three values following the peak of the distribution f(s), i.e. the interval s ∈ [N + 1, N + 2, N +3].

Using this quantitative description of the peaking behaviour, we wanted to demonstrate that the occurrence of these peaks was dependent on the presence of interactions in the experimental system and in the ASM. To test this for the LFP data we exchanged trials with each other within each sampling site. This way of shuffling the data kept the event statistics for each channel relative to the onset of a trial but destroyed the effects of interaction between the channels, which would be necessary for putative self-organized criticality. With this test we wanted to show that the distribution f(s) calculated from the unshuffled LFP data of M1 had a more extreme drop δ at s = N than the f_shuffled_(s) calculated from the shuffled data. To apply a similar test to the ASM – in order to prove that the appearance of peaks in the subsampled ASM also depended on intact interactions of the sites – we sampled the activity of a subset of sampling sites s2 and separated the data into trials of the same length as the experimental trials. Using the above test logic we then wanted to test whether f(s) calculated from topplings sampled on subset s2 of the critical ASM had a more extreme drop δ than the f_shuffled_(s) calculated from data sampled on the same subset of sampling sites of the ASM. This would prove that the appearance of peaks in our subsampled avalanche distributions depended on intact local interactions. We also tested whether the experimental results f(s; LFP, M1) fitted better with the f(s) calculated from unshuffled data from s2 of the ASM than with the f_shuffled_(s; ASM, s2) to assess whether the similarity between experimental data and model data depended on interaction rather than on single channel statistics. Again the drop δ was used as a measure for this match.

## Results

### Simulation Results

#### Avalanche distributions and branching parameters in the fully sampled models

In order to demonstrate that the models used in this study expressed SOC behaviour in the form of power law distributions and branching parameters near unity, we first present results from simulations of the fully sampled models.

**Figure 3:**
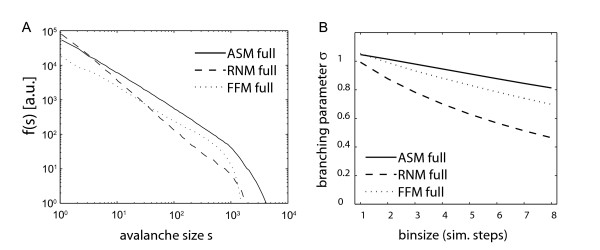
**Avalanche distributions and branching parameters for fully sampled model sytems**. (A) Avalanche Distributions for the fully sampled SOC models evaluated in logarithmic binning. The avalanche distributions of the fully sampled models do not change with the bin size, due to the infinite separation between subsequent avalanches in the models. All f(s) follow a power law for s < 500. The steeper decay for large s is caused by the finite size of the models. Solid line – Abelian sandpile model (ASM). Dashed line – random neighbour model (RNM). Dotted line – forest fire model (FFM). Avalanche distributions have been set apart for better visibility by multiplication with a constant factor per curve. (B) Dependence of the branching parameter σ on the bin size. Sigma is near unity for small bin sizes. For larger bin sizes sigma decays due to the finite size of the models. Solid line – Abelian sandpile model (ASM). Dashed line – random neighbour model (RNM). Dotted line – forest fire model (FFM). Bin size given in simulation steps.

The Abelian Sandpile Model (ASM) is a well known SOC model which was proposed to simulate the dynamics of avalanches on a pile of sand (see [15,47,48] for the absence of SOC in real world sandpiles; but see also [35,49]). It is run on a two dimensional lattice and activity triggered by a single grain propagates over the lattice via next neighbour connections and forms avalanches. The avalanche size distribution of the ASM, f(s; ASM, full), resulted in a power law with exponent τ = 1.1 for the fully sampled system (figure 3A). The deviation of f(s; ASM, full) from a power law for large s was caused by finite size effects [e.g. [36,38]]. The avalanche distributions observed in the FFM and RNM followed a power law, too (figure 3A). The exponents of f(s; FFM, full) and f(s; RNM, full) were τ = 1.0 and τ = 1.4 for the FFM and RNM, respectively. Thus, all three simulated SOC models expressed a power law when fully sampled but each model had a different slope exponent τ.

In addition to a description of the avalanche distributions, the branching parameter σ is used to quantify the state of a dynamical system [45]. We calculated σ for the ASM as a function of the bin size T (figure 3B). In general, increasing the bin size has two distinct effects on σ in a branching process: *First*, for *infinite *branching processes it is trivial to show that

(1.7)

Thus, σ derived from a process with σ(T = 1) > 1 increases with larger bin size T, σ derived from a process with σ(T = 1) = 1 is unity for any bin size, and σ derived from a process with σ(T = 1) < 1 decreases with larger bin sizes. *Second*, dissipative systems of *finite *size express finite avalanches, because avalanches that reach the boundaries are affected by dissipation mechanisms and tend to die out within finite time. Consequently, any branching parameter approaches zero for large bin sizes T:

(1.8)

In the critical ASM, σ was near unity for small bin sizes and decreased with a larger bin size (figure 3B) due to finite size effects (see equation (1.8)). The branching parameter of the FFM and RNM showed the same qualitative behaviour (figure 3B), although the finite size effects were stronger in the FFM and RNM, leading to a steeper decrease.

#### Avalanche distributions in subsampled critical models

Frequently, complex dynamical systems like the brain cannot be sampled completely due to experimental constraints. To assess the effects of subsampling in SOC systems, we implemented subsampling in the models by defining subsets of sampling sites and only sampled the activity that occurred on these specified subsets (figure 1B). To address the question whether the subsampling effects observed in SOC models were model-specific, we simulated three different SOC models, the ASM, the RNM and the FFM, and evaluated their activity on three different subsets of sampling sites.

##### SOC systems exhibit strong deviations from power law behaviour under subsampling conditions

**Figure 4:**
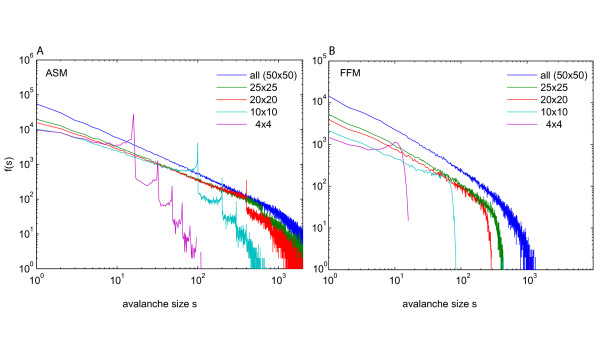
**Approximation of a power law distribution with increasing coverage of the system when subsampling**. Avalanche size distributions f(s) from models of grid size 50×50, sampled on centred, compact subareas of size 4×4 (purple), 10×10 (light blue), 20×20 (red), 25×25 (green) and fully sampled (blue). (A) ASM. (B) FFM. Note, how the characteristic subsampling effects vanish for the ASM (peaks) and the FFM (peak, steep drop off) with increasing coverage of the system.

In figure 1A (left), avalanche distributions were plotted which were calculated from activity of the ASM sampled on s2 (figure 1B, left). The avalanche distributions resulted in a power law for small avalanches of size s < 10. Surprisingly, f(s) of the ASM sampled on s2 showed characteristic peaks at avalanche sizes that were equal to N, the number of sampling sites, and to multiples of N. These peaks were less pronounced for smaller bin sizes and more distinct for larger bin sizes. Peaks at multiples of N indicated that *all *sampled sites were preferentially activated together within a single avalanche. With increasing the number of sampling sites of the SOC models, f(s) successively approximated a power law (figure 4). In the ASM and FFM, sampling of about 25% of the models' sites was necessary to obtain a power law robustly, although the slope was still different from that of the fully sampled models.

##### Subsampling effects are model specific

While activity sampled on s2 of the ASM resulted in an f(s) with peaks at multiples of N (figure 1A, full lines), the activity of the RNM (with identical dynamics but different topology) sampled on s2 resulted in an avalanche distribution that followed a steadily decaying function (same figure, dashed lines). The avalanche distribution of the FFM sampled on s2, f(s; FFM, s2), showed a distribution less steep than that of the RNM, but had a strong decay as approaching N (same figure, dotted lines). These types of distributions, which result in many small and few large avalanches are characteristic for *fully *sampled *sub*critical systems. However, here the 'apparently subcritical' avalanche distributions were caused by subsampling of *critical *models. Taken together, we found that sampling the same subset of sampling sites on the three different SOC models revealed substantially different avalanche distributions.

##### Subsampling effects depend on subsampling geometry

The avalanche distribution of the ASM changed from the shape with peaks at multiples of N for sampling s2 to a continuously decaying function for sampling s5 (figure 1A, full lines). Only a small step at N could be recognized in these latter distributions. This indicates that the appearance of the peaks depended strongly on the distance *d *between the sampling sites. Along similar lines, the FFM yielded more small avalanches and less large avalanches when sampled on s5 instead of s2 (same figure, dotted lines), but the differences between the distributions of s2 and s5 were not as pronounced as for the ASM.

##### Subsampling effects depend on the location of subsampling sites

The above results from the ASM and FFM were obtained on subsets of sampling sites that were centred with respect to the model geometry. In contrast, the subset c2 of sampling sites was situated next to a corner of the grid where dissipation mechanisms were strongest for the ASM. The distance between the sampled sites in subset c2 was 2 g.u., i.e. identical to that of subset s2. Due to enhanced dissipation of grains at the borders next to c2, the avalanche distribution calculated from activity sampled on c2 of the ASM showed fewer large avalanches than that sampled on s2, and f(s; ASM, c2) expressed no peaks. For small avalanches, s < <N, however, f(s) was very similar for s2 and c2 (figure 1A, right, full lines). The opposite was found for sampling c2 instead of s2 in the FFM (figure 1C, right, dotted lines). Fires could reach c2 only from two directions and thus trees had more time to accumulate next to the corners before they were set on fire. This facilitated dense clusters of trees and caused the bump in f(s) of the FFM at s ~11.

The distributions for the RNM sampled on area s2 were very similar to those sampled on both, c2 and s5 (figure 1A, dashed lines), as expected. Due to the random nature of the connections between the sites of the RNM, s2 is equivalent to c2 (both have N = 14 sites) and very similar to s5 (N = 15).

#### σ in subsampled critical systems

We next took a look at the apparent branching parameter σ in the SOC models to see whether it changed under subsampling conditions, and to evaluate whether these changes might be specific for models and subsampling areas.

##### The apparent branching parameter σ changes under subsampling conditions

As presented above, in all SOC models σ calculated from activity sampled on the *complete *grid was around unity for small bin sizes and decreased with larger bin size due to finite size effects (figure 3B). The branching parameter sampled on the tested subsets of sampling sites (s2, s5, c2) of the three SOC models was always smaller than unity (figure 1D, black lines). However, σ initially increased monotonically with larger bin size T, approaching a maximum. After reaching this maximum, finite size effects started to reduce the apparent branching parameter. This was expected as increasing the bin size increases the fraction of avalanches with a duration of 1 bin size. These particular avalanches have a single step branching ratio of zero and consequently σ decreases.

##### The apparent branching parameter depends on the distance between sampling sites

Similar to results obtained for the avalanche distributions under subsampling, the apparent branching parameter changed with distance *d *between the sampling sites for the ASM and the FFM (figure 1D, compare s2 and s5). The larger the distance *d *for these models, the smaller the apparent branching parameter at small temporal bin sizes – as expected, because larger distances *d *increase the probability that avalanches die out on their way from one sampled site to the next. Avalanches need around *d *simulation steps to reach the next sampling site [41]. Hence, with larger distances the time to reach the next sampling site may be longer than the applied window of temporal binning. Then a larger fraction of avalanches will contribute a single step branching ratio of zero.

##### The apparent branching parameter depends on the location of sampling sites

The apparent branching parameter also changed when the sampling sites were moved to the edges of the ASM or FFM (figure 1D, compare s2 and c2) – in line with the results for the respective avalanche distributions. For the RNM, the apparent branching parameter did not depend on the sampling site distance *d *and only weakly on the number of sites sampled. This behaviour had also been found for the avalanche distributions of this model.

##### Relation of apparent avalanche distributions and apparent branching parameters

Although the apparent branching parameter in our simulations was always smaller than unity, it showed larger values for those subsets of sampling sites which had 'apparently supercritical' avalanche distributions (s2, ASM; c2, FFM), and smaller values for those which showed 'apparently subcritical' avalanche distributions (s5, ASM and FFM; all subsets of sampling sites of the RNM).

#### Avalanche Distributions and branching parameters from LFP data

We now present the results obtained for the avalanche distributions and branching parameters in LFP recordings. These recordings inevitably come from extremely sparsely sampled systems. Hence, the avalanche distributions and branching parameters presented here should be compared to those obtained in the *subsampled *SOC models from the previous section.

##### LFP avalanche distributions deviate from power law behaviour and may express characteristic peaks

The avalanche distribution obtained from LFPs recorded in M1, f(s; LFP, M1) approximately followed a power law for s < 10. However, at multiples of N, the total number of electrodes evaluated, f(s; LFP, M1) expressed characteristic peaks (figure 1C, left). The peaks became more pronounced with larger bin size. After each multiple of N, f(s; LFP, M1) showed a strong drop δ of one or more orders of magnitude.

The LFPs in M2 were recorded with an inter electrode distance (IED) of 1.0 mm instead of 0.5 mm in M1 (as indicated in figure 1B middle). This resulted in an avalanche distribution which did not show the characteristic peaks observed in f(s; LFP, M1). Instead, f(s; LFP, M2) followed distributions between a power law and an exponential decay (also see additional file 1 for a presentation of all these data in log-linear coordinates).

The avalanche distribution obtained from LFPs from M3, sampled with an IED of 0.5 mm like that of M1, displayed a power law in the initial interval that was similar to f(s; LFP, M1) (figure 1C left and right, respectively). However, unlike f(s; LFP, M1), f(s; LFP, M3) did not result in peaks but showed a smooth transition into a steep decay when approaching s = N. For larger bin sizes the slope of this latter decay was more gentle again.

We determined the similarity between the distributions obtained from the LFPs and the distributions obtained from the different subsets of sampling sites on the three SOC models using the dissimilarity factor DF as defined in equation (1.4). A low DF is associated with a high similarity of the two distributions. Results for the similarity between distributions obtained from LFP data and models are listed in table 1. Distributions obtained from the ASM always provided a better fit to the experimental data than distributions obtained from the other models. More specifically, f(s; LFP, M1) was reproduced best by f(s; ASM, s2), that is by sampling s2 on the ASM. f(s; LFP, M2) and f(s; LFP, M3) were most similar to f(s; ASM, s5) obtained from sampling s5 on the ASM. We also observed a better match between the distributions of the LFP data from all three monkeys and of the *subsampled *ASM than for any match between the distributions of the LFP data and the *fully sampled *ASM.

**Table 1:** Dissimilarity Factor DF for avalanche distributions from in vivo data and SOC models

	s2	c2	s5	fully sampled
LFP M1 – ASM	**2.42**	2.64	2.56	3.99
LFP M1 – RNM	7.23	6.97	6.88	3.57
LFP M1 – FFM	6.15	5.90	5.98	4.00
				
LFP M2 – ASM	1.79	1.59	**0.82**	2.43
LPF M2 – RNM	6.75	6.58	6.60	1.86
LFP M2 – FFM	7.21	7.24	6.71	2.45
				
LFP M3 – ASM	2.28	1.79	**1.41**	3.07
LFP M3 – RNM	6.78	6.55	6.52	2.51
LFP M3 – FFM	6.74	6.76	6.30	3.09

Dissimilarity factor DF as defined in equation (1.4) between avalanche distributions from LFP data (M1, M2, M3) and models (ASM, RNM, FFM). Each lists a specific comparison between model and in vivo data. Each column contains the results fro a specific (sub-)sampling scheme (s2, c2, s5, full sampling). Best matching results are highlighted in BOLD face.

##### Branching Parameters from LFP Data

σ calculated from the binary LFP events was plotted in figure 1D, green lines. σ was largest, but still smaller than unity, for activity recorded in M1, which also showed the 'apparently supercritical' avalanche distribution. In comparison, the LFP data recorded in M2 and M3 resulted in a smaller σ, in agreement with their 'apparently subcritical' avalanche distributions. σ-values calculated from binary LFP events of M1 and M2 were most similar to σ-values calculated from activity of the subsets s2 and s5, respectively. s2 and s5 were the same subsets of sampling sites of the ASM which had already produced the best match to the avalanche distributions calculated from LFP data in M1 and M2.

#### Subsampling phenomena were dependent on the interaction of the systems' constituents

**Figure 5:**
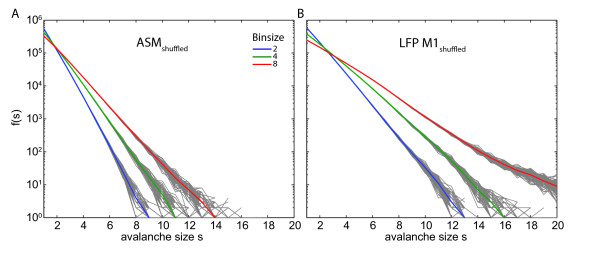
**Avalanche size distributions f(s) for shuffled data**. Avalanche size distribution f(s) obtained from trial shuffled data plotted in semi-logarithmic coordinates. Here, straight lines indicate exponential distributions and not a power law. Coloured lines are averages over 1000 different shufflings. Grey lines around the coloured lines are results obtained for single shufflings. (A) ASM; bin sizes: 2 (purple), 4 (green), 8 (red). (B) LFP data from M1; bin sizes: 2 (purple), 4 (green), 8 (red).

In order to assess whether the observed subsampling effects were caused specifically by interactions of the systems' constituents rather than event statistics at single sites, we used shuffled data in which temporal correlations between activities at the different sites of models or experimental recordings were destroyed (figure 5).

f(s; ASM, s2) calculated from topplings sampled on s2 had a more extreme drop δ than the f_shuffled_(s; ASM, s2) calculated from the shuffled data of the same subset of sampling sites (p < 0.001). This demonstrates that the occurrence of peaks in the event size distributions of the SOC systems tested here depends on intact interactions between the constituents of the system and not on the resulting event statistics at single sites (figure 5). Note that event statistics at *single *sites were indistinguishable for unshuffled and shuffled data by definition.

We found that the distributions f(s; LFP, M1) had a more extreme drop δ than the f_shuffled_(s; LFP, M1) calculated from the shuffled data (p < 0.001), indicating that the occurrence of peaks in the distribution f(s; LFP, M1) obtained from the LFP data in Monkey 1 depended on intact interactions between neuronal populations at the different recording sites (figure 5).

Furthermore, the experimental results f(s; LFP, M1) fitted better with the model results f(s; ASM, s2) calculated from unshuffled data than with the f_shuffled_(s; ASM, s2) calculated from the shuffled data (p < 0.001), suggesting that similar mechanisms may generate the peaks both in the model and the experimental data.

## Discussion

We studied the effects of subsampling in a controlled way in simulated SOC models and then assessed to what extent specific deviations from the expected power law behaviour obtained from *in vivo *LFP recordings may be attributable to subsampling effects. Our simulations demonstrated that subsampling of SOC systems typically results in avalanche distributions and branching parameters that strongly deviate from those expected for fully sampled SOC systems. Strikingly similar avalanche distributions were found in the LFP recordings.

### How many sampling sites are necessary to observe a power law distribution in a SOC system?

In our models, the avalanche distributions obtained upon subsampling strongly deviated from a power law. However, the avalanche distributions approximated a power law when increasing the number of sampling sites (figure 4). For the ASM and FFM, approximately 25% of the system had to be sampled to obtain a power law. The peaks in the avalanche distributions from LFP data clearly demonstrate that brain activity was sampled too sparsely to observe a power law if it were present, although we do not know how many electrodes at what distance would sufficiently sample brain activity. The fact that we do not need full sampling to observe approximate power laws, however, leaves hope that we can investigate SOC behaviour in the brain by massively increasing the number of sampled sites. Note, however, that the value of 25% obtained from subsampling the ASM can only be seen as an extremely rough estimate of the necessary coverage when dealing with *in vivo *recordings.

### Avalanche distributions of subsampled SOC models can show multiple peaks

The observed avalanche distributions in the simulated models strongly depended on the available sampling of the system. The avalanche distributions f(s; ASM) of the ASM, for example, showed the characteristic power law when the system was fully sampled (figure 3A). However, upon subsampling, f(s; ASM) depended strongly on the geometry of the sampled subset of sites and on the temporal bin size T applied.

For a small compact subset of sampling sites (s2), f(s;ASM, s2) showed peaks at N, the total number of sites sampled, and at multiples of N (figure 1A, left). This finding can be explained in a straight forward manner: In the ASM the avalanches tend to be compact [41]. Hence, there is an increased probability that large avalanches which hit the subset of sampling sites will also run over the entire subset. Activation of the entire subset of sampling sites is particularly probable if the area spanned by the subset is small compared to the total area affected by a large avalanche. In addition, avalanches in the ASM can express multiple waves [50]. These multiple waves in large avalanches then cause the peaks at *multiples *of the total number of sampled sites. In this respect, the detection of multiple peaks in an avalanche distribution obtained from *experimental data *may hint at an interaction topology and a dynamics of the experimental system under investigation that together enable the generation of compact, recurrent avalanches. Observation of multiple peaks in data recorded from the brain *in vivo *therefore suggests the existence of compact, recurrent avalanches of neuronal activity.

Multiple peaks in f(s) have already been described for a *fully *sampled, *supercritical *model [37], and are considered to indicate supercriticality. However, we showed that *critical *systems, too, may exhibit distributions with multiple peaks. These peaks, interpreted naively, would lead to a classification of the system as supercritical. We suggest to use the term 'apparently supercritical' for these distributions obtained when subsampling a SOC model. Our results demonstrate that subsampling provides a second, and independent, mechanism for generating avalanche distributions with one or multiple peaks. Therefore, any observation of peaks in avalanche distributions from subsampled experimental data cannot be seen as an indicator of supercriticality.

### The shape of avalanche distributions depends on the geometry of the subsampling scheme

As described above, an 'apparently supercritical' f(s) was found in the critical ASM when the distance between the sampling sites was small (s2). For larger distances between the sampling sites (s5), f(s) showed a reduced frequency for large avalanches compared to a power law distribution (figure 1A, middle). This kind of avalanche distribution f(s) is characteristic for the *sub*critical state of *fully *sampled systems [e.g. [37]]. In the subsampled model systems these 'apparently subcritical' distributions f(s) are generated when sampling from a subset of sampling sites with large distances between the sites, because then the sampled sites are distributed over a larger area and the avalanches of the ASM are rarely large enough to affect all sites of a subset. This causes a decreased probability of observation for large avalanches under this particular subsampling geometry and results in an apparently subcritical f(s).

Emergence of both, apparently *sub*critical and apparently *super*critical distributions f(s) upon subsampling of the same critical model, suggests that applying multiple independent sampling schemes with different resolutions may improve our knowledge about the state a system under investigation considerably and may help to prevent misclassifications.

### Avalanche distributions depend on the position of the subset of sampling sites in the system

We observed a distribution f(s) with multiple peaks when sampling the ASM on the compact subset s2. Sampling on c2, which had the same geometry, but was located next to a corner of the grid, resulted in an avalanche distribution with fewer large avalanches than the distribution sampled on s2, and did not express any peaks (figure 1A, right, solid lines). This observed suppression of peaks and large avalanches in the distribution for c2 was caused by a strong impact of dissipation (effects of dissipation are also referred to as finite size effects in SOC literature). Dissipation was larger in c2 than in s2 because c2 was located next to a corner of the grid, bordering two dissipating edges, while s2 was located in the middle of the grid. Our results demonstrate that f(s) in the ASM obtained upon subsampling did not only depend on the geometry, but also on the location of the subset of sampling sites. Consequently, sampling at different locations in an experimental SOC system in which not all elements are equivalent may yield different avalanche distributions upon subsampling, even if the geometry of the recording device remains the same.

By subsampling the ASM, we demonstrated that the absence of a power law under subsampling conditions is not sufficient evidence to reject the hypothesis that the system is in a critical state. Therefore, false classifications of the state of a system may occur under subsampling conditions. We propose to vary the geometry and location of the subsampling area in experimental systems that cannot be fully sampled, since f(s) may depend strongly on these factors.

### Subsampling effects are system specific

Since the ASM showed very specific subsampling effects, we asked whether other SOC models with different dynamics, like the FFM, and different connectivity, like the RNM, may also express subsampling effects, and whether these subsampling effects were model specific. Indeed, we found that avalanche distributions depended on both, the internal dynamics, and the topology of connections between the system's constituents.

### Dependence of subsampling effects on internal dynamics

We compared two systems with identical topology but different dynamics – the ASM and the FFM: In the ASM, the sand grains added to a site come mainly from its next neighbours, while in the FFM trees are distributed to random sites, accumulate and then contiguous tree clusters can burn down. This has various effects on the avalanche characteristics: While sand avalanches in the ASM are compact, forest fires are not compact because the tree distribution is patchy. Hence, the area affected by a fire includes usually a large fraction of empty sites. This difference in the dynamics affects the avalanche distribution observed under subsampling: As a consequence of the patchy tree distribution in the FFM it is rare that all sites of a subset of sampling sites (not to be confused with a cluster of trees) burn down since, first, all sampling sites of the subset must be covered by a tree, and, second, all of these sites have to belong to the same cluster. Note that subsampling is typically not compact and that trees on a subset of sampling sites can belong to different clusters. Therefore not even all trees on a subset necessarily burn down in a single fire. Due to these effects of the patchy tree distribution, the avalanche distribution f(s) from the FFM on subset s2 did not show the apparently supercritical avalanche distribution, which had been observed when sampling the same subset on the ASM.

There is, however, a way to generate apparently supercritical distributions with increased probability for large fires via subsampling of the FFM: f(s) sampled on c2, located next to a corner, showed an increased probability for fires of size s~0.8 N (N = number of subsampling sites). Fires tended to be larger on c2 than on s2, because near the corners of the grid fires can reach the tree cluster only from two sides, and trees have more time to accumulate before they are burnt down. Therefore, clusters on c2 tended to be larger on average.

In sum, both models, the ASM and the FFM showed apparently supercritical avalanche distributions, but the mechanisms leading to the apparently supercritical f(s) are different. As a consequence of the different mechanisms, the shapes of the two distributions were also different.

Subsampling had already been applied to the FFM [36]. However, in that study, the subsampling areas were relatively large, containing at least 400 sites. In contrast, we applied sparse subsampling, analyzing the activity of 16 sites at most. Only upon sparse subsampling, the specific subsampling effects described above occur. When more and more sites were taken into account, the avalanche distribution continuously approached a power law (figure 4).

### Dependence of subsampling effects on the topology of connections

In contrast to the behaviour of the ASM and the FFM upon subsampling, neither the configuration nor the location of the sampling sites mattered for the avalanche distributions of the RNM. This is intuitively plausible because the neighbours which receive the grains during an avalanche are chosen anew in every time step and, hence, spatial structure is meaningless for the RNM. Nevertheless, we still observed subsampling effects in the RNM: f(s) depended on the total number of sampled sites and showed an 'apparently subcritical' avalanche distribution when subsampled (figure 1A). We note that in models with next neighbour connections like the ASM and the FFM the distribution f(s) strongly depended on the subsampling geometry, while this was not the case for models with random dynamic connections, where the concept of next neighbours has no meaning. In the RNM, the avalanche distribution depended only on the number of sampled sites.

In our *experimental *LFP recordings, f(s) depended on the location and inter-electrode distance of the subsampling area. This suggests that the next neighbour propagation of activity could play an important role in the statistics of neuronal network activity. In line with this hypothesis, the self organized critical FFM and ASM accounted better for the activity patterns observed in the LFP data than the RNM.

### Subsampling affects the apparent branching parameter

When systems capable of SOC behaviour are fully sampled, changes in their state from critical to sub- or supercritical are sensitively reflected in the value of their branching parameter σ. Here, we wanted to know whether the branching parameter σ observed *under subsampling *would also truthfully reflect the unchanged true state of a system or whether σ would strongly change under subsampling, despite the unchanged state. We also asked whether σ observed under subsampling would exhibit similar apparent sub- and supercriticality as the corresponding avalanche distributions for the subsampled models did.

When fully sampled, all three SOC models that we used in our simulations showed a σ of approximately unity (figure 3B), as expected for systems in a critical state. With larger bin size T, σ decreased due to finite size effects as described in equation (1.8). Upon subsampling, σ was always smaller than unity (figure 1D), in contrast to the results obtained for full sampling. Under subsampling, σ initially increased with larger T because avalanches which appeared on a single site of a subset of sampling sites needed several time steps to reappear on a different site later. Increasing the bin size thus concatenated these fractions of an avalanche and led to an increased branching parameter. For even larger bin sizes, finite size effects (equation (1.8)) became dominant and σ decreased under subsampling conditions, as it did for full sampling conditions.

### Relation of apparently sub- and supercritical avalanche distributions to the observed branching parameters

Interestingly, σ was largest when those subsets were sampled which showed an 'apparently supercritical' avalanche distribution f(s) (s2 of the ASM and c2 of the FFM, figure 1D). Still, the observed σ under subsampling was smaller than unity even when 'apparently supercritical' f(s) were observed. A σ smaller than unity is typical for subcritical systems when they are fully sampled. Hence, we call a σ smaller than unity under subsampling conditions an 'apparently subcritical' σ. Our simulation results suggest that the observation of an apparently *sub*critical branching parameter σ together with an apparently *super*critical avalanche distribution in an experimental system is a strong indicator that the results in question might have been affected by subsampling. Additional recordings, employing different recording geometries and more recording sites, seem warranted in this case to avoid false classifications of the state of the system. Since σ is an easily obtained measure and since it depends in a non-linear way on the internal dynamics and topology of the system under investigation, it adds important information on the dynamics of a system and should be evaluated together with f(s) in any criticality analysis.

### Using model specific subsampling effects for model selection

The differences in f(s) and σ upon subsampling in the three SOC models investigated here were caused by the different underlying model dynamics and interaction topologies: The RNM has no stable next neighbour connections which govern the FFM and the ASM dynamics. In addition, the FFM differs from the ASM in that it allows only for a single expansion of an avalanche, while the ASM can create two or more waves within a single avalanche [50]. As a consequence, we found that effects of sparse subsampling not only depended on the geometry and location of the subset of sampling sites, but on the specific dynamics of the SOC models as well. Despite investigating only SOC models here, we propose that f(s) and σ may be particularly useful to test any set of physiologically motivated models of brain function that is supposed to model available experimental data [e.g. [51,52]]. This is because the apparent avalanche distribution f(s) and branching parameter σ obtained upon subsampling are highly complex measures of a system's topology and dynamics. Nevertheless, f(s) and σ are easily obtained from the data. The representation of complex properties of a system, the sensitivity to system dynamics and the computational simplicity, make f(s) and σ particularly useful to constrain degrees of freedom in physiologically plausible models, and to gain a better insight into the underlying dynamics of the recorded data, beyond the analysis of criticality proper.

### How do avalanche distributions and branching parameters from in vivo recordings compare to modelling results?

To date, simultaneous recordings of electrophysiological data are still constrained to relatively few electrodes, given the huge number of interacting circuits in the brain. Hence, when analyzing data from these recordings, we may deal with a heavily subsampled system and subsampling effects may play an important role.

We found that the avalanche distributions calculated from up to 16 simultaneously recorded LFP channels did not show a power law (figure 1C). This behaviour was expected in the light of our results from subsampling SOC models if the brain indeed operated in a critical state. Importantly, avalanche distributions f(s) from the ASM, but not from the FFM and RNM reproduced the experimental findings best (table 1). These best matching avalanche distributions were obtained using subsampling geometries in our models that resembled the electrode configuration in our LFP recordings (i.e. square sampling geometries as opposed to rectangular or linear ones). In addition, we searched for the distance *d *in the subsampling geometry on the ASM that would result in the most similar avalanche distribution for each of the experimentally obtained avalanche distributions f(s). We found that subsets of sampling sites with small *d *in the ASM best matched f(s) obtained from LFP recordings with small inter-electrode distance, and subsets with large *d *best matched f(s) obtained from LFP recordings with large inter-electrode distance.

These findings have various implications: First, various models of SOC exist, and these differ quite strongly when subsampled. Consequently, it is not enough to ask whether the brain might be in a critical state, but it would be more precise to ask, in which critical state the brain might be, since different groups of SOC systems exist (for renormalization groups see [53,54]). Second, the dynamics of the ASM and those of LFPs are expected to have features in common which are not present in the FFM or RNM. Probably, the dynamics of LFP activity are dominated by next neighbour propagation of activity, and might exhibit multiple activations of a single site during a single avalanche, because these were the prominent features of the ASM that led to the observed specific subsampling effects. Furthermore, the observation of 'apparently *super*critical' distributions f(s) in combination with 'apparently *sub*critical' branching parameters in both, the ASM and the LFP data, suggests that avalanches in both systems are generated by similar mechanisms.

While we demonstrated that the ASM might have more features in common with the LFP activity than the other SOC models investigated here, the ASM is still not a physiologically plausible model for LFP activity. Models which resemble LFP activity better and in addition have the properties of SOC are to be developed for a better understanding of LFP dynamics, and for assessing the potential existence of a critical state in the brain.

### Differences between avalanche distributions observed in the three monkeys

Avalanche distributions from M1 and M2 differed with respect to the observation of peaks that were only found in M1. This difference can be well explained by the increased inter electrode distance in M2 and is in line with predictions from modelling (figure 1). The avalanche distributions from M1 and M3 also differed and we observed no peaks in M3, despite the fact that the geometry of the recording grid was identical to M1. However, electrodes in M3 were placed in a different cortical area (dorsolateral prefrontal cortex in M3, ventral prefrontal cortex in M1 and M2). A different connectivity in this area may have led to the diverging results.

Interestingly, avalanche distributions from M2 and M3 both did not show peaks, although data from M3 were recorded with the small electrode distance. This finding nevertheless is compatible with our findings in the ASM, where we showed that avalanche distributions can be similar, even when sampled with different distances between sites (figure 1A, s5 and c2). The underlying mechanisms leading to the similar f(s) despite of different electrode distances can be manifold, however. We think that the most probable reason is perhaps that the electrodes were located in different areas (dorsolateral and ventral PFC). In addition, we currently do not know whether the brain is in an SOC state at all, and whether the cortex can be considered as one large system or whether we should consider it as being composed of smaller relatively independent units. Hence, another possible explanation of our findings would be that our electrodes in M3 were placed close to the edge of such a module and we indeed observed finite size effects.

In sum, we would only like to stress that the finding of similar avalanche distributions despite different electrode distances does not exclude that the brain is in a critical state

### Comparison to in vivo results from the anaesthetized developing brain

In a recent study Gireesh and Plenz investigated the occurrence of neuronal avalanches in the developing rat cortex both *in vitro *and *in vivo *[4]. They observed power law statistics in their *in vivo *data while we did not observe a power law, raising the question about the reason for this difference in results. There are several differences between the study of Gireesh and Plenz and our study that make a direct comparison difficult. First, Gireesh and Plenz obtained their data from the developing cortex while we measured in the adult animal. Second, Gireesh and Plenz used at least 32 electrodes for their recordings, while we could evaluate only up to 15 channels. A larger number of sampling sites reduces subsampling effects (figure 4). However, even with their 32-site recordings, f(s) showed a peak at s = 32 (their figure 1E). These peaks suggest, that subsampling affects their data and their observed power law exponent might not be the same in the fully sampled system (compare our figure 4). Third, the anaesthetic Urethane used by Gireesh and Plenz acts on GABA, Glycin and Nicotinic receptors as well as on a large variety of other ion channels and interacts with other neuro-pharmacological agents [55]. In addition, Urethane may lead to damage in the developing brain [56]. Hence, the alteration of neuronal and circuit function is possibly profound and we think that the use of anaesthesia is possibly the most important factor behind the differences between the two studies.

### Peaked avalanche distributions: previous experimental observation

In the past it has often been tacitly assumed that a sufficient fraction of an experimental system was sampled to classify its state unambiguously, ignoring possible subsampling effects. A notable exception is the study by Beggs and Plenz who found a power law distribution for population spike avalanches sampled from slice cultures of rat cortex *in vitro *in simultaneous recordings with 60 electrodes [2,32]. For these recordings, they investigated the effects of subsampling by evaluation of the activity sampled on a half or a quarter of the electrodes. Although the authors do not specifically comment on this, their avalanche distributions for compact subsets of sampling sites showed a small peak at the total number of electrodes used for the evaluation. These peaks were more pronounced the smaller the subset of sampling sites was (figure 3c in reference [32]). With larger distances between the sites, the peaks vanished (figure 3d in reference [32]). Both, an increase of peak height with a smaller compact set of sampling sites, and a disappearance of the peaks with larger distances between the sites, is in full agreement with the behaviour we observed when subsampling the critical ASM, and when evaluating LFP activity recorded in the awake monkey. Thus, we interpret these peaks observed in f(s) of the slice culture recordings by Beggs and colleagues as a first experimental observation of this specific kind of subsampling effect. Their results also demonstrate that an increase of the number of recording sites leads to a better approximation of a power law for f(s) in an experimental system that probably is in the critical state. This is in accordance with the behaviour of all three SOC models, where an increase of sampling sites leads to a continuous approximation to a power law for the avalanche distribution (figure 4).

### The role of local interactions for inter event interval and avalanche distributions

**Figure 6:**
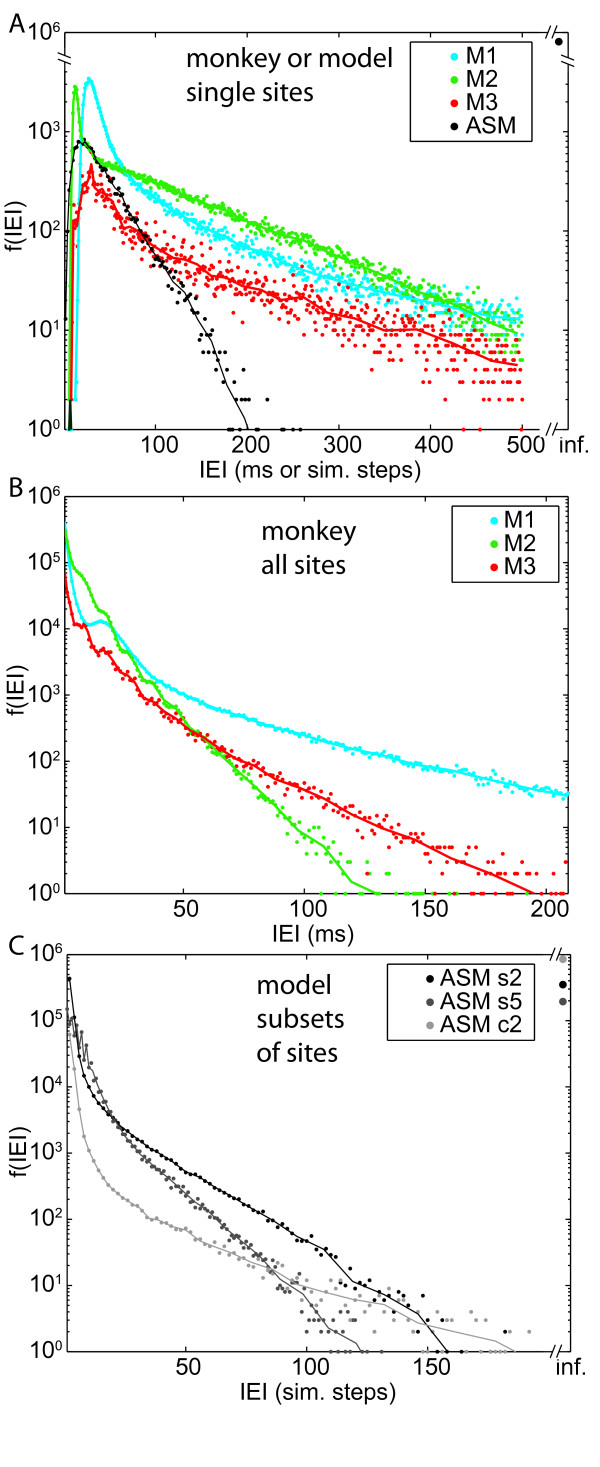
**Distribution of inter event intervals (IEIs)**. (A) IEIs evaluated for data obtained from *a single site *in M1 (olive), M2 (dark green), M3 (light green), and of the ASM (black). (B) IEIs evaluated for the data obtained from *all electrodes *in M1 (olive), M2 (dark green), and M3 (light green). (C) IEIs evaluated for data obtained by subsampling the ASM on s2 (black), s5 (dark grey), and c2 (light grey).

In our SOC models, there exist only three possible inter event intervals (IEI) [e.g. [2,31]] when the system is *fully *sampled. During an avalanche, the IEIs are always zero or one simulation steps, and between subsequent avalanches, the IEI is infinity (infinite separation of timescales). Evaluating the ASM on a *single site *only, however, reveals that the IEI distributions of each site have exponential tails like Poisson processes even in the critical models (figure 6A). Hence, single site statistics that approximate Poisson statistics in time (IEIs) are fully compatible with SOC behaviour that is organised spatio-temporally. Due to the approximately poissonian single site statistics, destroying the multivariate dependency between the sites of the model system results in the expected exponential distribution of avalanche sizes, both in experimental and model data (figure 5).

In a critical system the processes on connected sites are extremely dependent on each other, however, due to the local interactions. Therefore, when looking at the IEI distribution calculated for events across all electrodes or all sites of a subset, we see a deviation from an exponential distribution (figure 6B, C): We find convex curves in the log-linear plots, indicating a surplus of both, extremely small and extremely large IEI compared to the exponential distribution. Therefore we expect that the IEI distribution calculated for any sufficiently large number of sites of a SOC system will not be exponential. However, the f(IEI) evaluated from very few sampling sites in a SOC system may be similar to that of independent Poisson processes. However, observing an exponential IEI distribution upon sparse subsampling is not sufficient to state that the system is not SOC.

### Comparing the experimentally obtained σ for LFP recordings to the model σ upon subsampling

The branching parameter σ is a well defined and easily obtained measure in any dynamical system with binary events. We calculated σ for the various toppling dynamics of the models as well as for the binary events extracted from the LFP activity (figure 1D). Similar to results from subsampling the ASM, we found that σ obtained from the LFP recordings was smaller than unity for small bin sizes T and increased with larger T. In addition, we found that the experimentally obtained σ was most similar to that obtained from subsampling the ASM on s2 and s5 for recordings from M1 and M2, respectively. This is in agreement with the similarities between the respective avalanche distributions from the experiments and those obtained from the models upon subsampling (table 1). However, a further increase of the bin size T led to σ > 1 for recordings from all three monkeys (additional file 2). This is in contrast to the findings in the critical models upon subsampling, since none of the subsets of sampling sites showed σ > 1 upon subsampling. However, Beggs and Plenz found a similar behaviour for the branching parameter σ calculated from the population spike activity of their slice culture preparations (figure 7D in reference [2]): σ increased with larger bin sizes T and exceeded unity for T > 4 ms. The increase of the apparent branching parameter obtained from our experimental data and from slice culture recordings by Beggs and Plenz [2] above a value of unity may either be explained by subsampling effects not present in the specific critical models investigated here, or it might suggest that the brain is rather in a supercritical state, since supercriticality implies a larger branching parameter, and consequently, an apparent branching parameter larger than unity might be expected in supercritical systems even upon subsampling. However, further investigations, comparing subsampling effects in critical, subcritical and supercritical models are necessary to distinguish better these three states in experimental systems that can not be fully sampled.

### Does the brain operate in a SOC state?

Several features which are common in SOC systems are indeed found in measures of brain activity *in vivo*. Nevertheless, we are still a long way from deciding whether an experimental system under investigation truly qualifies as SOC. Apart from our limited knowledge about the behaviour of SOC measures in subsampled systems, other fundamental problems exist for the case of the brain. Jensen stated that SOC can be expected in slowly driven, interaction dominated threshold (SDIDT) systems, and that SOC systems should show a separation of time scales, that is, the relaxation process (i.e. the avalanche) should be much faster than the drive to the system [17] – otherwise the dynamics would be dominated by the external drive. While a separation of time scales is easily implemented in models like the ASM, the distinction between drive applied to the system and its internal relaxation dynamics is far from being clear for the case of brain activity, even at a conceptual level. Hence, it is extremely difficult to assess whether the concept of an SDIDT system capable of a critical state is applicable to brain activity at all. Rigorous tests for the various defining features and ingredients of a SOC system like separation of time scales, cooperativity and the self-organizing property need to be developed. At a practical level, the complex and non-linear scaling of the avalanche distribution and the branching parameter upon subsampling poses additional challenges to the correct classification of the operating state of a system.

## Conclusion

Neither a power law nor a branching parameter of unity are necessarily observable in subsampled SOC systems. This may ultimately render efforts to establish the presence or absence of *critical *self-organized behaviour in brain function futile when relying on these properties of SOC systems alone. Nevertheless, subsampling effects are highly specific for the connection topology and for the dynamics of the local interactions of the system under investigation. In addition, changing subsampling effects by varying the subsampling geometries provides rich additional information. Thus, measures derived from SOC system theory like branching parameters and avalanche distributions provide information on complex system properties in a computationally efficient, compact and non-trivial way, and are useful beyond the scope of SOC theory. The subsampling geometry can be used as an experimentally accessible additional free parameter to gain further information. We suggest to use measures from the theory of SOC systems as powerful constraints to select between various models proposed for a certain real-world system under investigation. In this regard, the extremely specific subsampling effects observed in the *in vivo *data may help to establish which features of model SOC systems are 'brain-like', assisting us to constrain models of brain function in future research. On the experimental side, future research using massively parallel recordings with hundreds of electrodes will be necessary to fully understand subsampling effects in detail and to exploit this knowledge to foster our understanding of brain function.

## Authors' contributions

MJHM conceived of the idea to investigate the distribution of neuronal avalanches obtained from in vivo data with respect to SOC behavior. VP detected the peaks in the avalanche distributions from these experimental data, and conceived of the idea that these peaks were due to subsampling effects. VP also simulated the SOC model systems. MW and VP worked on the theoretical foundations of subsampling effects. MW provided the environment for the large-scale simulation of SOC models, defined the similarity factor and devised the statistical tests for the investigation of the drop in the avalanche distributions. VP and MW drafted the manuscript. MJHM conceived of the behavioural paradigm, performed the monkey training and electrophysiological recordings, and supervised raw data preparation. All authors read and approved the final manuscript.

## Supplementary Material

Additional file 1 **Supplementary Figure S1 – Subsampling effects in logarithmically binned avalanche distributions in model systems and in vivo LFP data**. **Data as in figure **1** but presented in log-linear coordinates**. This figure provides the same results as figure 1, but plotted in log-linear instead of double logarithmic coordinates. (A) Avalanche distributions f(s) calculated from events sampled on the respective subsets of sampling sites as given in (B). Avalanche distributions of the ASM (full lines), the RNM (dashed lines) and the FFM (dotted lines) are plotted. The colours indicate the different bin sizes (blue 2 time steps; green 4 time steps; red 8 time steps). (B) Recording electrode configurations and subsampling areas used in simulations. The circles indicate the position of the electrodes, full circles indicate the electrodes that provided data for the evaluation of the LFPs. The inter electrode distance is given at the bottom of each figure. The full circles at the same time indicate the configuration of the subset of sampling sites sampled in the models. The left part of each figure indicates the position of the sampling sites with respect to the grid the model was simulated on. The left figure displays the subset of sampling sites s2: 4×4 sites with distance 2 grid units (g.u.) between the sites, located in the center of the grid. The middle figure displays the subset of sampling sites s5, and the right figure the subset of sampling sites c2. (C) Avalanche size distributions f(s) over avalanche size s for the binary events calculated from the LFPs. The colours indicate the bin size (blue 2 ms; green 4 ms; red 8 ms): left figure – Monkey 1 (LFP M1), middle – Monkey 2 (LFP2 M2), right – Monkey 3 (LFP M3). The corresponding electrode configurations are plotted in part (B). Click here for file

Additional file 2 **Supplementary Figure S2 – Branching parameter σ for large bin sizes**. **The branching parameter σ for bin sizes larger than presented in the main manuscript**. Branching parameter σ over bin size s (in ms for LFP data and steps for simulated data) for the subsampled models and the LFP data. (A) σ for sampling on subset s2 of sampling sites. Green, solid line: σ for LFP of M1; dashed lines: σ for the subsampled ASM, FFM, and RNM; (B) σ for sampling on subset s5 of sampling sites for the models and the LFP of M2; same colour codes; (C) σ for sampling on subset c2 of sampling sites for the models and the LFP of M3. The difference between the experimental and the simulation results for large bin sizes is probably due to the infinite time between subsequent avalanches in the model, while the time between the neuronal avalanches in vivo is rather short. Please note the logarithmic scale of the x-axis. Click here for file

## References

[B1] Shimono M, Owaki T, Amano K, Kitajo K, Takeda T (2007). Functional modulation of power-law distribution in visual perception. Phys Rev E Stat Nonlin Soft Matter Phys.

[B2] Beggs JM, Plenz D (2003). Neuronal avalanches in neocortical circuits. J Neurosci.

[B3] Beggs JM, Plenz D (2004). Neuronal avalanches are diverse and precise activity patterns that are stable for many hours in cortical slice cultures. J Neurosci.

[B4] Gireesh ED, Plenz D (2008). Neuronal avalanches organize as nested theta- and beta/gamma-oscillations during development of cortical layer 2/3. Proc Natl Acad Sci USA.

[B5] Freeman WJ (2005). A field-theoretic approach to understanding scale-free neocortical dynamics. Biol Cybern.

[B6] Linkenkaer-Hansen K, Nikouline VV, Palva JM, Ilmoniemi RJ (2001). Long-range temporal correlations and scaling behavior in human brain oscillations. J Neurosci.

[B7] Poil S-S, van Ooyen A, Linkenkaer-Hansen K (2008). Avalanche dynamics of human brain oscillations: relation to critical branching processes and temporal correlations. Hum Brain Mapp.

[B8] Linkenkaer-Hansen K, Monto S, Rytsälä H, Suominen K, Isometsä E, Kähkönen S (2005). Breakdown of long-range temporal correlations in theta oscillations in patients with major depressive disorder. J Neurosci.

[B9] Carandini M (2004). Amplification of trial-to-trial response variability by neurons in visual cortex. PLoS Biol.

[B10] Fontanini A, Katz DB (2008). Behavioral states, network states, and sensory response variability. J Neurophysiol.

[B11] Arieli A, Sterkin A, Grinvald A, Aertsen A (1996). Dynamics of ongoing activity: explanation of the large variability in evoked cortical responses. Science.

[B12] Frette V, Christensen K, Malthe-Sørenssen A, Feder J, Jøssang T, Meakin P (1996). Avalanche dynamics in a pile of rice. Nature.

[B13] Davidsen J, Paczuski M (2005). Analysis of the spatial distribution between successive earthquakes. Phys Rev Lett.

[B14] Baiesi M, Paczuski M (2004). Scale-free networks of earthquakes and aftershocks. Phys Rev E Stat Nonlin Soft Matter Phys.

[B15] Bak P, Tang C, Wiesenfeld K (1987). Self-organized criticality: An explanation of the 1/f noise. Phys Rev Lett.

[B16] Binney JJ, Dowrick NJ, Fisher AJ, Newman MEJ (1992). The Theory of Critical Phenomena.

[B17] Jensen HJ (1998). Self-Organized Criticality.

[B18] Harris T (1963). The Theory of Branching Processes (Dover Books on Advanced Mathematics).

[B19] Beggs JM (2007). Neuronal Avalanche. Scholarpedia.

[B20] Beggs JM (2008). The criticality hypothesis: how local cortical networks might optimize information processing. Philos Transact A Math Phys Eng Sci.

[B21] Hsu D, Chen W, Hsu M, Beggs JM (2008). An open hypothesis: is epilepsy learned, and can it be unlearned?. Epilepsy Behav.

[B22] Schoner G, Kelso JA (1988). Dynamic pattern generation in behavioral and neural systems. Science.

[B23] Makarenkov VI, Kirillov AB (1991). Self-organized criticality in neural networks. Applications of Artificial Neural Networks II: 1991.

[B24] Worrell GA, Cranstoun SD, Echauz J, Litt B (2002). Evidence for self-organized criticality in human epileptic hippocampus. Neuroreport.

[B25] Haldeman C, Beggs JM (2005). Critical branching captures activity in living neural networks and maximizes the number of metastable States. Phys Rev Lett.

[B26] Bak P (1996). How nature works: the science of self-organized criticality.

[B27] Germana J, Lancaster R (1995). Brain dynamics, psychophysiological uncertainty and behavioral learning. Integr Physiol Behav Sci.

[B28] Anderson CM, Holroyd T, Bressler SL, Selz KA, Mandell AJ (1993). 1/F- like Spectra in Cortical and Subcortical Brain Structures: A Possible Marker for Behavioral State-Dependent Self-Organisazion. AIP Conf Proc.

[B29] Beggs JM (2008). The criticality hypothesis: how local cortical networks might optimize information processing. Philos Transact A Math Phys Eng Sci.

[B30] Bertschinger N, Natschläger T (2004). Real-time computation at the edge of chaos in recurrent neural networks. Neural Comput.

[B31] Bedard C, Kroeger H, Destexhe A (2006). Does the 1/f frequency scaling of brain signals reflect self-organized critical states?. Phys Rev Lett.

[B32] Plenz D, Thiagarajan TC (2007). The organizing principles of neuronal avalanches: cell assemblies in the cortex?. Trends Neurosci.

[B33] Gisiger T (2001). Scale invariance in biology: coincidence or footprint of a universal mechanism?. Biol Rev Camb Philos Soc.

[B34] Newman MEJ (1997). A Model of Mass Extinction. Journal of Theorectical Biology.

[B35] Liu, Jaeger, Nagel (1991). Finite-size effects in a sandpile. Phys Rev A.

[B36] Schenk K, Drossel B, Clar S, Schwabl F (2000). Finite-size effects in the self-organizedd critical forest-fire model. Eur Phys J B.

[B37] Eurich CW, Herrmann JM, Ernst UA (2002). Finite-size effects of avalanche dynamics. Phys Rev E Stat Nonlin Soft Matter Phys.

[B38] Drossel (2000). Scaling behavior of the Abelian sandpile model. Phys Rev E.

[B39] Stumpf MP, Wiuf C, May RM (2005). Subnets of scale-free networks are not scale-free: sampling properties of networks. Proc Natl Acad Sci USA.

[B40] Drossel, Schwabl (1992). Self-organized critical forest-fire model. Phys Rev Lett.

[B41] Dhar D (2006). Theoretical studies of self-organized criticality. Physica A.

[B42] Pettersen KH, Devor A, Ulbert I, Dale AM, Einevoll GT (2006). Current-source density estimation based on inversion of electrostatic forward solution: effects of finite extent of neuronal activity and conductivity discontinuities. J Neurosci Methods.

[B43] Pettersen KH, Einevoll GT (2008). Amplitude variability and extracellular low-pass filtering of neuronal spikes. Biophys J.

[B44] Wu W, Wheeler DW, Staedtler ES, Munk MH, Pipa G (2008). Behavioral performance modulates spike field coherence in monkey prefrontal cortex. Neuroreport.

[B45] Harris TE (1963). The theory of branching processes.

[B46] Kullback S, Leibler RA (1951). On information and sufficiency. Annals of Mathematical Statistics.

[B47] Held, Solina, Keane, Haag, Horn, Grinstein (1990). Experimental study of critical-mass fluctuations in an evolving sandpile. Phys Rev Lett.

[B48] Rosendahl J, Vekic M, Kelly J (1993). Persistent Self-Organization in Sandpiles. Phys Rev E.

[B49] Jaeger, Liu, Nagel (1989). Relaxation at the angle of repose. Phys Rev Lett.

[B50] Ivashkevich EVK DV, Priezzhev VB (1994). Waves of topplings in an Abelian sandpile. Physica A.

[B51] El Boustani S, Pospischil M, Rudolph-Lilith M, Destexhe A (2007). Activated cortical states: experiments, analyses and models. J Physiol Paris.

[B52] Markram H (2006). The blue brain project. Nat Rev Neurosci.

[B53] Zhang YC (1989). Scaling theory of self-organized criticality. Phys Rev Lett.

[B54] Bagnuls C, Bervillier C (1990). Field-theoretical approach to critical phenomena. Phys Rev B Condens Matter.

[B55] Hara K, Harris RA (2002). The anesthetic mechanism of urethane: the effects on neurotransmitter-gated ion channels. Anesth Analg.

[B56] Thompson KW, Wasterlain CG (2001). Urethane anesthesia produces selective damage in the piriform cortex of the developing brain. Brain Res Dev Brain Res.

