# Supplementary figures and images for: Subsampling effects in neuronal avalanche distributions recorded in vivo
Source: BMC Neurosci. 2009 Apr 29;10:40. doi: 10.1186/1471-2202-10-40 (PMC2697147; doi:10.1186/1471-2202-10-40)

A

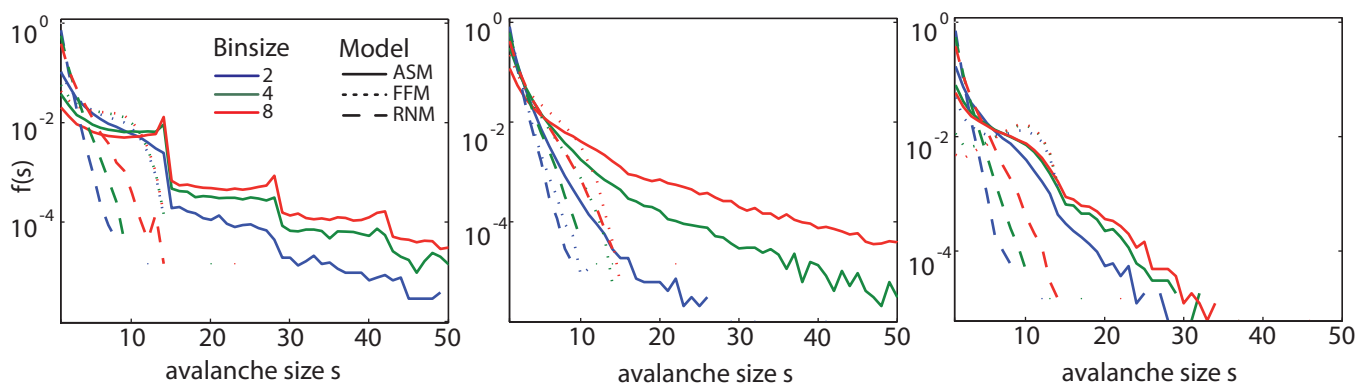

B

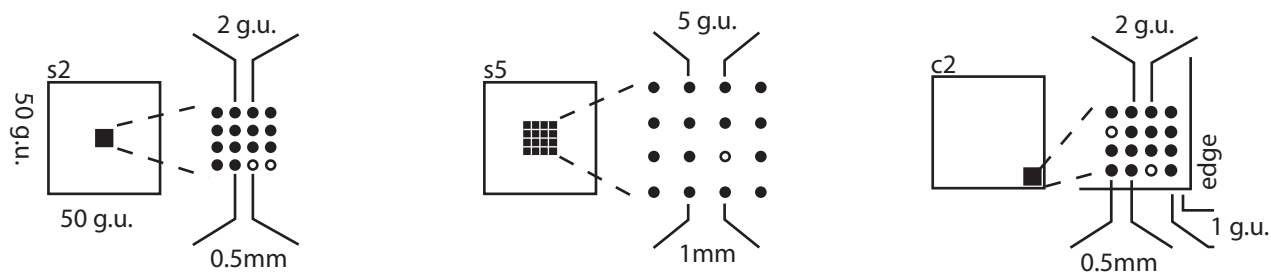

C

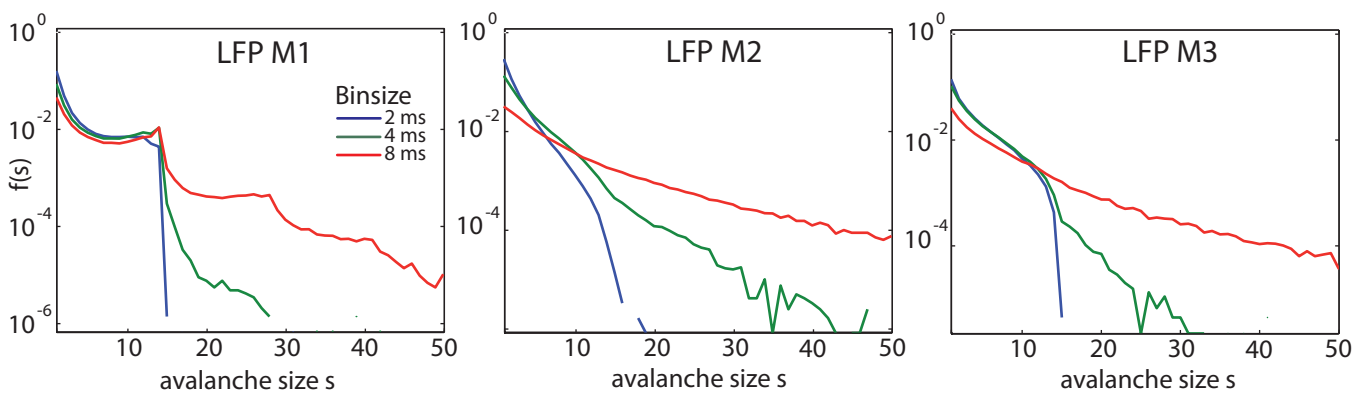

Supplement: Additional file 1 — Supplementary Figure S1 – Subsampling effects in logarithmically binned avalanche distributions in model systems and in vivo LFP data. Data as in figure 1 but presented in log-linear coordinates. This figure provides the same results as figure 1, but plotted in log-linear instead of double logarithmic coordinates. (A) Avalanche distributions f(s) calculated from events sampled on the respective subsets of sampling sites as given in (B). Avalanche distributions of the ASM (full lines), the RNM (dashed lines) and the FFM (dotted lines) are plotted. The colours indicate the different bin sizes (blue 2 time steps; green 4 time steps; red 8 time steps). (B) Recording electrode configurations and subsampling areas used in simulations. The circles indicate the position of the electrodes, full circles indicate the electrodes that provided data for the evaluation of the LFPs. The inter electrode distance is given at the bottom of each figure. The full circles at the same time indicate the configuration of the subset of sampling sites sampled in the models. The left part of each figure indicates the position of the sampling sites with respect to the grid the model was simulated on. The left figure displays the subset of sampling sites s2: 4×4 sites with distance 2 grid units (g.u.) between the sites, located in the center of the grid. The middle figure displays the subset of sampling sites s5, and the right figure the subset of sampling sites c2. (C) Avalanche size distributions f(s) over avalanche size s for the binary events calculated from the LFPs. The colours indicate the bin size (blue 2 ms; green 4 ms; red 8 ms): left figure – Monkey 1 (LFP M1), middle – Monkey 2 (LFP2 M2), right – Monkey 3 (LFP M3). The corresponding electrode configurations are plotted in part (B). [file 1471-2202-10-40-S1.pdf]

A

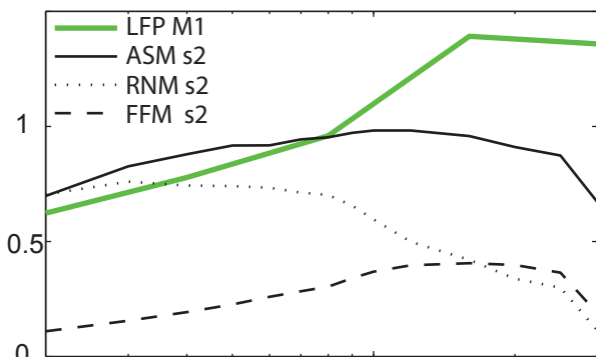

B

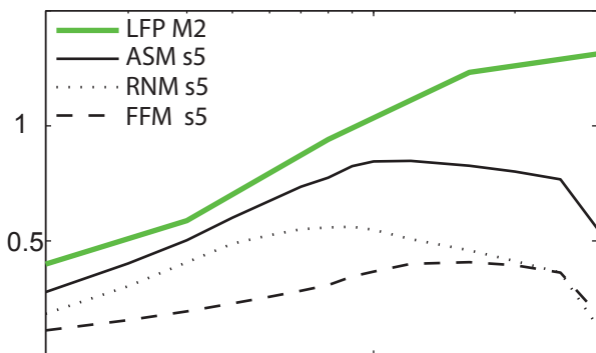

C

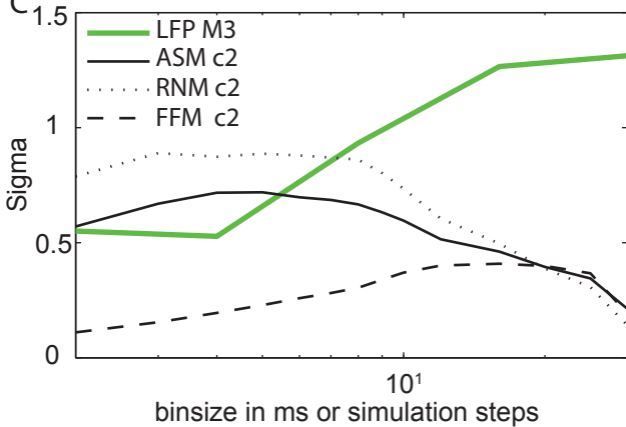

Supplement: Additional file 2 — Supplementary Figure S2 – Branching parameter σ for large bin sizes. The branching parameter σ for bin sizes larger than presented in the main manuscript. Branching parameter σ over bin size s (in ms for LFP data and steps for simulated data) for the subsampled models and the LFP data. (A) σ for sampling on subset s2 of sampling sites. Green, solid line: σ for LFP of M1; dashed lines: σ for the subsampled ASM, FFM, and RNM; (B) σ for sampling on subset s5 of sampling sites for the models and the LFP of M2; same colour codes; (C) σ for sampling on subset c2 of sampling sites for the models and the LFP of M3. The difference between the experimental and the simulation results for large bin sizes is probably due to the infinite time between subsequent avalanches in the model, while the time between the neuronal avalanches in vivo is rather short. Please note the logarithmic scale of the x-axis. [file 1471-2202-10-40-S2.pdf]
